# Article on Dentition

**Published:** 1883-11

**Authors:** A. H. Good

**Affiliations:** Selma, Ind.


### Article on Dentition.

BY DR. A. H. GOOD, SELMA, IND.

[Read in the Section on Diseases of Children, June, 1883.]

*Mr. President and Gentlemen of the American Medical Association:* The process of dentition is not properly classed as a disease, but the diseases which accompany it are numerous; hence I have given my paper the nomenclature Dentition. In order to be brief, I will not refer to statistics. In dentition, with its accompanying diseases, the mortality is generally greater than in all other diseases to which children are subjected. Some children are more easily disturbed by teething than others, because of not being so strongly organized, or because of some peculiar susceptibility to its influence. I conceive it to be true that the process of dentition acts more severely (although a natural one) than would foreign bodies similarly located. At the extremity of each tooth-root is the dental foramen through which the dental nerve passes, and during the growth of the tooth there is an inflammatory action, which, coming through the nerve agency, reflects with great power through the same channel, and is generally distributed through the sympathetic nerves. We then have, in addition to the tooth acting as a foreign body, a reflex nervous irritability. Our attention is first called to the teeth, and when the gums are swollen they should be divided, to relieve pressure, pain, and inflammatory action.

When apthous ulceration occurs, it should be treated with a solution of persulphate of iron, or some other astringent lotion.

We have, as a concomitant, a functional derangement of the stomach and bowels, resulting from innervation, the sequel of the reflex nervous irritation, and displaying a yeasty and soured condition. This we *may* find, upon microscopic investigation, to contain myriads of bacteria. Can we not, then, trace the origin of bacteria, if found in the stomach and bowels of of these patients, to be the result of mal-nutrition and the cause of cholera infantum? The treatment for this condition varies according to the mildness or severity of each individual case and the surrounding circumstances (viz. foul or pure air, squalid or comfortable apartments, and a strict observance of the laws of hygiene). But when the disease is established, and the removal impracticable or impossible, then comes the severe trial to the physician, anxious parents, and suffering child. A high rate of mortality, every act of the physician closely watched, even his changes of countenance from anxiety to forlorn hope closely scrutinized, and so "ad infinitum." The thermal ranges are various in different and even in the same cases, in the acute form often reaching 103, 104, or 105 degrees Fahrenheit; in the more progressive form usually much lower. The pulse generally corresponds to the temperature.

Viewing the disease from my standpoint, I begin the treatment for the disease proper with nervines, and as a normal temperature (or a somewhat elevated temperature, is a favorable condition for micro-organisms and inflammation), I use cold compresses to the bowels and ice water injections, and for the secondary symtoms I use pepsine, sub. nit. of bismuth, and carbolic acid.

DISCUSSION ON DR. GOOD'S PAPER.

Dr. Woodworth, of Illinois, said that in a practice of thirty-three years he has always been in the habit of lancing the swollen gums of teething children, with good results. Besides giving relief, he thought the teeth came on more readily with scarification of the gums than without.

Drs. Earle, of Chicago, and Boothby, of Wisconsin, testified to the relief given in the reflex diarrhœa of teething children by scarifying the gums, medicine being entirely withheld, but particular attention paid to diet. Dr. Boothby never scarified only for a diarrhœa, believing that to be the only indication.

